# Competitive Metabolism of Polycyclic Aromatic Hydrocarbons (PAHs): An Assessment Using In Vitro Metabolism and Physiologically Based Pharmacokinetic (PBPK) Modeling

**DOI:** 10.3390/ijerph19148266

**Published:** 2022-07-06

**Authors:** Jordan N. Smith, Kari A. Gaither, Paritosh Pande

**Affiliations:** 1 Biological Sciences Division, Pacific Northwest National Laboratory, Richland, WA 99352, USA; kari.gaither@pnnl.gov (K.A.G.); paritosh.pande@pnnl.gov (P.P.); 2 Department of Environmental and Molecular Toxicology, Oregon State University, Corvallis, OR 97331, USA

**Keywords:** benzo[a]pyrene, dibenzo[def,p]chrysene, Relative Potency Factor approach, mixture risk assessment

## Abstract

Humans are routinely exposed to complex mixtures such as polycyclic aromatic hydrocarbons (PAHs) rather than to single compounds, as are often assessed for hazards. Cytochrome P450 enzymes (CYPs) metabolize PAHs, and multiple PAHs found in mixtures can compete as substrates for individual CYPs (e.g., CYP1A1, CYP1B1, etc.). The objective of this study was to assess competitive inhibition of metabolism of PAH mixtures in humans and evaluate a key assumption of the Relative Potency Factor approach that common human exposures will not cause interactions among mixture components. To test this objective, we co-incubated binary mixtures of benzo[a]pyrene (BaP) and dibenzo[def,p]chrysene (DBC) in human hepatic microsomes and measured rates of enzymatic BaP and DBC disappearance. We observed competitive inhibition of BaP and DBC metabolism and measured inhibition coefficients (*K_i_*), observing that BaP inhibited DBC metabolism more potently than DBC inhibited BaP metabolism (0.061 vs. 0.44 µM *K_i_*, respectively). We developed a physiologically based pharmacokinetic (PBPK) interaction model by integrating PBPK models of DBC and BaP and incorporating measured metabolism inhibition coefficients. The PBPK model predicts significant increases in BaP and DBC concentrations in blood AUCs following high oral doses of PAHs (≥100 mg), five orders of magnitude higher than typical human exposures. We also measured inhibition coefficients of Supermix-10, a mixture of the most abundant PAHs measured at the Portland Harbor Superfund Site, on BaP and DBC metabolism. We observed similar potencies of inhibition coefficients of Supermix-10 compared to BaP and DBC. Overall, results of this study demonstrate that these PAHs compete for the same enzymes and, at high doses, inhibit metabolism and alter internal dosimetry of exposed PAHs. This approach predicts that BaP and DBC exposures required to observe metabolic interaction are much higher than typical human exposures, consistent with assumptions used when applying the Relative Potency Factor approach for PAH mixture risk assessment.

## 1. Introduction

Polycyclic aromatic hydrocarbons (PAHs) are common environmental contaminants consisting of fused benzene rings. Incomplete combustion of organic compounds from fires, volcanic activity, and burning fossil fuels generate different PAHs [1]. As a result of their global formation, PAHs are found throughout the environment in soil, air, and water. Humans are exposed to complex mixtures of PAHs from various environmental sources on a daily basis. Human exposures to PAHs vary with personal activity and location, generally ranging from 1 to 17 µg/d [2,3,4,5]. In non-occupational settings, up to 70% of PAH exposure for non-smoking humans is associated with diet and the oral route of exposure [6,7]. Primary dietary sources of PAHs include cereals, oils, vegetables, and food cooked over an open flame [8].

Some PAHs are procarcinogens, and the United States Environmental Protection Agency (US EPA) frequently assesses PAH mixture cancer risk using the Relative Potency Factor approach [9]. The Relative Potency Factor approach estimates cancer risk of a chemical mixture by summing doses of component mixture compounds after normalizing each component dose with a relative cancer potency of that component compared to an index compound [9]. For PAH mixtures, EPA uses benzo[a]pyrene (BaP) as the index compound [9]. Application of the Relative Potency Factor approach assumes that each component has a similar toxicological action and that interactions among mixture components do not occur at typical human exposures (i.e., PAHs exhibit additive effects) [9]. These required assumptions make risk assessment of a “whole mixture” a preferable alternative approach to the Relative Potency Factor approach; however, for practical broad-purpose application, the Relative Potency Factor approach is frequently used to assess risk of PAH mixtures.

Since many PAHs require metabolic activation to form the ultimate toxicant, metabolism is one process in which competitive interactions among PAHs found within a mixture can occur, potentially violating a key assumption of the Relative Potency Factor approach. Once absorbed in the body, cytochrome P450 enzymes (CYPs) oxidize protoxicant PAHs, initiating bioactivation or detoxification pathways. For example, CYPs can transform BaP to reactive metabolites such as BaP-7,8-dihydrodiol-9,10-epoxide capable of biding with DNA, forming DNA adducts, causing mutations, and eventually manifesting as cancer [10,11]. CYPs, glutathione transferases (GSTs), or uridine 5′-diphospho-glucuronosyltransferase (UGTs) can contribute to the detoxification of BaP and/or BaP metabolites by forming BaP-dihydrodiols, -diones, hydroxylated metabolites, or Phase II conjugates, thereby enabling BaP elimination [12,13,14]. Specific enzymes can contribute to bioactivation, detoxification, or both pathways. For example, Sulc et al. demonstrated via Supersomes expressing human CYPs that CYP1A1 and CYP2C19 produced 96% of BaP-7,8-diol (a precursor to BaP-7,8-dihydrodiol-9,10-epoxide), initiating a bioactivation pathway [14]. In the same experiment, CYP3A4 produced 53% of 3-hyroxy-BaP, a detoxified metabolite of BaP [14]. CYP1A1 also produced a significant amount of 3-hydroxy-BaP (27%), demonstrating that some CYPs can initiate the bioactivation and detoxification pathways.

Some enzymes can metabolize multiple PAHs, leading to mixtures of PAHs competing as substrates for the same enzyme and competitive inhibition at certain internal concentrations of PAH mixtures. For example, researchers have reported that CYP1A1, CYP1B1, and other enzymes can bioactivate many different PAHs [11,15,16]. Researchers have also observed the inhibition of PAH metabolism in mixed enzyme systems. Nichols et al. observed metabolic inhibition of binary PAH mixtures including phenanthrene, pyrene, and BaP in hepatic S9 fraction from trout [15]. In bacteria, Stringfellow and Aitken observed competitive inhibition of metabolism among naphthalene, methylnaphthalenes, and fluorene [16]. Mixture interactions can affect adverse effects of PAHs. Rice et al. observed increased formation of BaP DNA adducts in mouse skin and increased formation of diol epoxide metabolites following combined exposures of fluoranthene, pyrene, and BaP [17]. These observations suggest that the same enzyme can metabolize multiple PAHs, competitive inhibition of PAH metabolism occurs in mixture exposures, and increased toxicity may result from PAH mixture exposures.

The objective of this study was to evaluate competitive inhibition of metabolism of PAH mixtures in humans. Altering specific enzymatic pathways of PAHs could have direct consequences on rates of bioactivation or detoxification and, ultimately, human risk. Additionally, the Relative Potency Factor approach assumes that common PAH human exposures will not cause interactions among PAH mixture components at typical human exposures. To evaluate this objective, we measured inhibition coefficients (*K_i_*) of binary mixtures of PAHs using BaP and dibenzo[def,p]chrysene (DBC), a PAH with a Relative Potency Factor of 30, in human hepatic microsomes [9]. We developed a physiologically based pharmacokinetic (PBPK) interaction model by integrating two PBPK models of DBC and BaP with metabolism interaction terms [18,19]. This allowed us to account for the observed competitive inhibition of parent PAH metabolism in in vitro metabolism assays and to predict the internal dosimetry of these compounds. We used the interaction PBPK model to simulate different PAH exposures to humans to identify what conditions would cause changes in internal dosimetry of DBC or BaP. Finally, we measured inhibition coefficients of Supermix-10, a mixture of the most abundant PAHs measured at the Portland Harbor Superfund Site, on BaP and DBC metabolism to compare the potency of DBC and BaP as inhibitors to environmental mixtures [20]. Results of this study will inform on the potential for competitive inhibition of PAH metabolism at relevant human exposures.

## 2. Materials and Methods

### 2.1. Chemicals

Acetone, ethyl acetate, acetonitrile, methanol, sodium sulfate, sulfuric acid, potassium phosphate salts (dibasic and monobasic), and phosphate buffered saline (PBS) were purchased from Fisher Scientific (Pittsburgh, PA, USA). Benzo[a]pyrene was purchased from Sigma-Aldrich (St. Louis, MO, USA). Reduced Nicotinamide adenine dinucleotide phosphate (NADPH) was purchased from MilliporeSigma (Burlington, MA, USA). All solvents used in this study were of high-performance liquid chromatography (HPLC) grade. Drs. Shantu Amin and Arun Sharma (Pennsylvania State University (State College, PA, USA)) synthesized dibenzo[def,p]chrysene according to previous methods [21,22,23].

Supermix-10 is a mixture of the top 10 PAHs found at the Portland Harbor Superfund site on a concentration basis including benzo(a)anthracene, retene, pyrene, phenanthrene, naphthalene, fluorene, fluoranthene, chrysene, acenaphthylene, and 2-methylnaphthalene (Table 1). EPA, International Agency for Research on Cancer (IARC), or American Conference of Governmental Industrial Hygienists (ACGIH) classified three PAHs within Supermix-10 as carcinogenic: benzo(a)anthracene, naphthalene, and chrysene (Table 1). Supermix-10 was synthesized as previously described [20].

### 2.2. In Vitro Metabolism Studies

Since human PBPK models exist for BaP and DBC [18,19] and pharmacokinetic data from these compounds also exist from microdosing studies in humans [24,25,26,27,28], we focused our metabolism studies on human liver microsomes. Pooled human liver microsome samples were obtained from Sekisui Xenotech, LLC (Kansas City, KS, USA). Pooled samples contained contributions from 200 individuals having equal male:female ratio and predominantly from humans between 19 and 78 years of age (3 individuals 10–18 years old).

Metabolism of BaP and DBC was measured using an in vitro metabolism assay, using conditions optimized previously in our laboratory [29,30,31]. Briefly, within 500 µL total volume, reactions consisted of MgCl_2_ (3 mM), microsomes (2.0 mg/mL), 0.1 M phosphate buffer (pH 7.4), and excess NADPH (1.5 mM). Control samples using inactivated microsomes absent of NADPH were also included. Microsomes were preactivated via 37 °C incubation on a thermal shaker for 5 min. Immediately following pre-activation, BaP (0.05 µM–2.5 µM) or DBC (0.025 µM–1 µM) dissolved in acetone (final incubation concentration: 0.4% by volume) was added to samples. Samples were incubated at 37 °C for 0–30 min or 0–60 min, respectively. Reactions were quenched by adding 500 µL 0.9 M H_2_SO_4_, and samples were immediately placed on ice until extraction. A standard curve was prepared (0.015 µM–25.0 µM) in inactivated microsomes. An internal standard was added to each quenched sample (DBC for BaP kinetics reactions and BaP for DBC kinetics reactions). Experiments were performed in triplicate.

### 2.3. Competitive Inhibition Metabolism Assays

Competitive inhibition of PAH metabolism was measured using conditions described above. Enzymatic reactions were initiated by adding a combination of a substrate (i.e., BaP or DBC) at a consistent concentration and a competitive inhibitor (i.e., BaP, DBC, or Supermix-10) at varying concentrations. For example, when evaluating inhibitors of BaP metabolism, BaP (0.14–0.18 µM initial concentration) was co-incubated with varying concentrations of DBC (0.1–10 µM) or Supermix-10 (0.1–30 µM). Likewise, when evaluating inhibitors of DBC metabolism (0.17 µM initial concentration), DBC was co-incubated with varying concentrations of BaP (0.1–10 µM) or Supermix-10 (0.1–30 µM). Incubations were terminated at 0–30 min for BaP and 0–60 min for DBC via addition of 500 µL 0.9 M H_2_SO_4_, and samples were placed on ice. An internal standard was added as described above; retene was used as the internal standard for BaP with DBC as the competitive inhibitor and vice versa. Each experimental condition (e.g., substrate, concentration, time point, etc.) was conducted in triplicate.

### 2.4. Analyte Quantitation

Analytes were extracted from samples with liquid–liquid extraction and measured using high-performance liquid chromatography (HPLC). First, BaP, DBC, or retene was added as an internal standard depending on incubations conditions being tested. Samples were extracted using three 500 µL volumes of ethyl acetate. Extracts were dried under a gentle stream of nitrogen and resolubilized in 500 µL methanol. Analytes were quantified by reverse phase HPLC using an Agilent 1260 Infinity HPLC system equipped with an Agilent G4290B variable wavelength detector and an Agilent G1321B SPECTRA fluorescence detector (Santa Clara, CA, USA). Sample (10 µL) was injected into an Ascentis 25 cm × 4.6 mm, 5 µm C18 column (Sigma-Aldrich, St. Louis, MO, USA). A gradient of water and acetonitrile from 55:45 to 0:100 was employed from 0 to 10 min and then was held at 100% acetonitrile until 22 min at a flow rate of 0.95 mL/min. Retene was measured via UV detection at 250 nm, while DBC and BaP were measured via fluorescence detection. Excitation and emission wavelengths were 230 nm and 430 nm, respectively. Elution times for compounds were roughly 21 min for DBC, and 18 min for BaP. Quantification was accomplished using a linear regression fit to an external calibration curve prepared in tandem with samples. The approximate limit of reliable quantification (LOQ) for both BaP and DBC was ~0.0015 μM.

### 2.5. Data Analysis

As with previous in vitro PAH metabolism studies conducted in our lab, metabolism rates were calculated based on substrate disappearance [29,30]. For each specific substrate and inhibitor concentration combination, metabolism rates (as initial rates of substrate disappearance; nmol/min/mg microsome) were calculated using an exponential regression of substrate concentrations (µM) (e.g., BaP or DBC) as a function of time (min). Confidence intervals of metabolism rates were calculated using a nonparametric bootstrap, where metabolism rates were resampled with replacement at each concentration, and the regression was repeated on the resampled dataset (*n* = 1000).

BaP and DBC incubations without any inhibitors demonstrated saturating metabolism rates, and we evaluated metabolism rates (*dS*/*dt*) as a function of substrate concentration ([*S*]) using three mathematical models: a Michaelis–Menten equation for saturable kinetics (Equation (1)) and two models describing scenarios where mixtures of low-capacity/high-affinity and high-capacity/low-affinity enzymes are active. These two models include a Michaelis–Menten clearance equation for non-saturable high-capacity/low-affinity enzymes (Equation (2)) and a double Michaelis–Menten equation for saturable high-capacity/low-affinity enzymes (Equation (3)). Models were optimized using a maximum likelihood objective. The Bayesian information criterion (BIC) was used to judge the best-fit model for evaluating metabolism. For Michaelis–Menten kinetics, intrinsic clearance values (*Cl_int_*) of the first order phase were estimated by dividing the *Vmax* by the respective *Km*. Fit parameter confidence intervals were calculated using a parametric bootstrap (*n* = 1000), where each best fit model was re-optimized to sampled metabolic rates assuming a normal distribution at each time point:

(1) $$\frac{dS}{dt}=\frac{Vmax\times \left[S\right]}{Km+\left[S\right]}$$

(2) $$\frac{dS}{dt}=\frac{Vmax_{1}\times \left[S\right]}{Km_{1}+\left[S\right]}+Cl_{int2}\times \left[S\right]$$

(3) $$\frac{dS}{dt}=\frac{Vmax_{1}\times \left[S\right]}{Km_{1}+\left[S\right]}+\frac{Vmax_{2}\times \left[S\right]}{Km_{2}+\left[S\right]}$$

Once we identified the baseline best-fit models for BaP and DBC incubations without inhibitors, we then evaluated additional models to describe the effects of varying concentrations of inhibitors. Without inhibitors, our approach identified the Michaelis–Menten-clearance equation for non-saturable high-capacity/low-affinity enzymes as the best-fit model (Equation (2)) for both BaP and DBC incubations (see Section 3.1). We modified this equation to account for competitive metabolism of concentrations substrate and inhibitor ([*I*]) described using an inhibition constant (*K_i_*). We considered two modifications to Equation (2), first competing with the high-affinity/low-capacity enzyme (Equation (4)) and second competing with the low-affinity/high-capacity enzyme (Equation (5)). As described above, we used the BIC to judge the best-fit model:

(4) $$\frac{dS}{dt}=\frac{Vmax_{1}\times \left[S\right]}{Km_{1}\times (\frac{1+\left[I\right]}{K_{i}})+\left[S\right]}+Cl_{int2}\times \left[S\right]$$

(5) $$\frac{dS}{dt}=\frac{Vmax_{1}\times \left[S\right]}{Km_{1}+\left[S\right]}+Cl_{int2}\times \left[S\right]\times (\frac{1+\left[I\right]}{K_{i}})$$

Software used to analyze in vitro metabolism data was “R: A language and environment for statistical computing”, version 4.0.3 (Vienna, Austria).

### 2.6. PBPK Interaction Model

**Figure 1:**
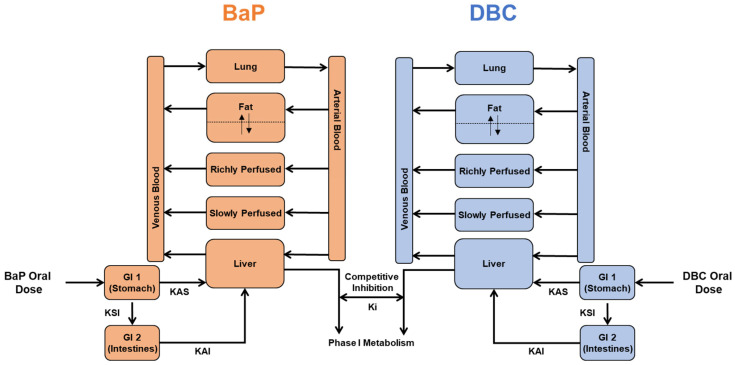
Schematic of a physiologically based pharmacokinetic (PBPK) interaction model for benzo[a]pyrene and dibenzo[def,p]chrysene (DBC). We modified previously published PAH models [18,19] to include competitive inhibition of the metabolism of both parent compounds (i.e., DBC inhibits BaP metabolism, and BaP inhibits DBC metabolism).

To understand implications of observed competitive inhibition of PAH metabolism, we developed a PBPK model to simulate effects of metabolic interactions between BaP and DBC in humans. A recently developed PBPK model for DBC in humans [19] was modified to include absorption, distribution, metabolism, and elimination pathways for BaP (Figure 1). Model equations in this interaction model for BaP and DBC were identical to the published DBC model [19], except for the metabolism rate equations for the parent compounds. To account for competitive inhibition between DBC and BaP, we used Equation (4) as the model that best fit data from our in vitro inhibition experiments (See Section 3.1). We used in vitro to in vivo extrapolation methods to translate metabolism constants measured here in human liver microsomes to appropriate scales in the model [19]. Intrinsic clearance and *Vmax* terms were scaled to liver volume using 30 mg microsomal protein per g of liver tissue [21,31,32]. All other model parameters (compartment volumes, blood flows, absorption rates, partitioning coefficients, etc.) were assumed to be of a reference human of 73 kg and the same for BaP and DBC, two similar, highly lipophilic compounds (log *Kow* 6.13 and 7.71, respectively) [19].

The interaction PBPK model simulated two scenarios: (1) a competitive metabolism between BaP and DBC (i.e., using measured *K_i_* values) and (2) no competitive metabolism (i.e., *K_i_* values set to 1 × 10^99^ µM, indicating that the inhibition of PAH metabolism requires a very high inhibitor concentration). For each scenario, the model simulated 16 oral doses of BaP and DBC ranging from 0.1 ng to 100 kg (increasing by a factor of 10) for a grand total of 256 exposure combinations.

We measured the effect of competitive interaction by the inhibitor PAH on substrate PAH as the ratio of the area under the curve (AUC) of each parent PAH concentration in blood over time simulated under scenario 1 (assuming competitive metabolism) to the corresponding AUC value obtained for scenario 2 (assuming no competitive metabolism). A resulting ratio of 1 therefore indicates no effect of the inhibitor PAH on the substrate PAH AUC, whereas a value >1 indicates significant metabolic inhibition by the inhibitor PAH on the substrate PAH, increasing the AUC for the substrate PAH.

The interaction PBPK model was coded and implemented in Magnolia, Version 1.2.2 (Magnolia Sciences, LLC, Orlando, FL, USA).

## 3. Results

### 3.1. In Vitro Metabolism

**Figure 2:**
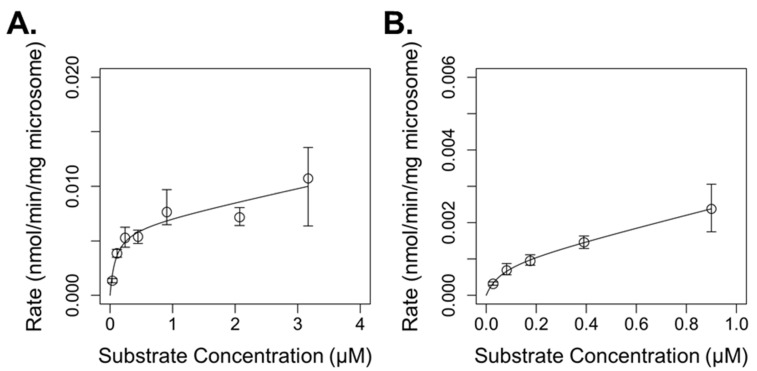
Rates of benzo[a]pyrene (BaP; (**A**)) and dibenzo[def,p]chrysene (DBC; (**B**)) disappearance in human hepatic microsomes at different substrate concentrations. Lines are best fit models describing the metabolism.

We observed enzymatic disappearance of BaP and DBC in pooled human liver microsomes in two distinct phases characteristic of multiple dominant enzymes. BaP metabolism exhibited a rapid first-order phase until 0.24 µM, shifting afterwards to a slower first-order phase to 3.3 µM, the highest substrate concentration tested (Figure 2A). Similarly, rates of DBC metabolism also demonstrated two phases, although rates of DBC metabolism were 3–5 times slower than rates of BaP metabolism (Figure 2B). After evaluating metabolism of BaP and DBC with three metabolism models, the Michaelis–Menten clearance model demonstrated the best fit for both substrates, as evidenced by the lowest BICs (Table 2). This suggests that two dominant enzymes influenced BaP and DBC metabolism in human microsomes depending on the substrate concentration: a high-affinity/low-capacity and low-affinity/high-capacity enzyme, and the best fit models offer estimates of the respective metabolism parameters of those enzymes (Table 3). The low-affinity/high-capacity enzyme did not saturate at BaP and DBC concentrations utilized here (Figure 2).

**Figure 3:**
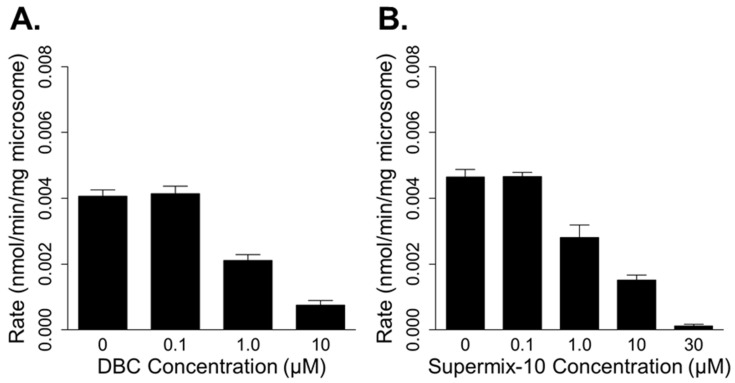
Rates of benzo[a]pyrene (BaP) metabolism (0.14–0.18 µM initial concentration) in human hepatic microsomes co-incubated with increasing concentrations of dibenzo[def,p]chrysene (DBC; (**A**)) or Supermix-10 (**B**).

Co-incubating DBC or Supermix-10 with BaP caused dose-dependent decreases in rates of BaP metabolism, suggesting competition among these PAHs for enzymes in human hepatic microsomes. DBC (≥1 µM) significantly inhibited (≥48%) the rate of BaP metabolism at an initial substate concentration of 0.14 µM BaP (Figure 3A). Likewise, 1 µM of Supermix-10 caused a 40% inhibition in BaP metabolism (0.18 µM BaP initial substrate concentration) (Figure 3B). Increased inhibitor concentrations in DBC and Supermix-10 caused further decreases in the rates of BaP metabolism (Figure 3). The BIC evaluation of competitive inhibition models indicated that inhibition constants modifying the Michaelis–Menten constant (*Km*_1_) of the first enzymatic phase (high-affinity/low-capacity enzyme) resulted in the best fit model (Equation (4)) (BIC −48 vs. −31 for DBC and −55 vs. −37 for Supermix-10). Confidence intervals (95%) of optimized inhibition constants (*K_i_*) for DBC and Supermix-10 overlapped, suggesting no statistical difference in inhibition potency of BaP metabolism (Table 4).

**Figure 4:**
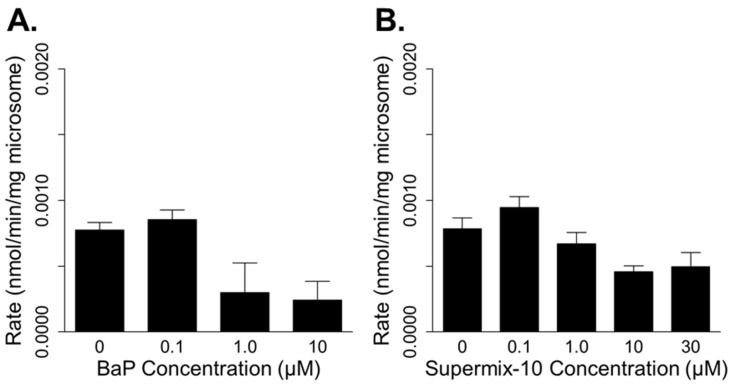
Rates of dibenzo[def,p]chrysene (DBC) metabolism (0.17 µM) in human hepatic microsomes co-incubated with increasing concentrations of benzo[a]pyrene (BaP; (**A**)) or Supermix-10 (**B**).

BaP and Supermix-10 inhibited DBC metabolism in a dose-dependent manner. Co-incubated BaP (1 µM) inhibited DBC metabolism by 62% at 0.17 µM substrate concentration (Figure 4A). Supermix-10 also inhibited DBC metabolism but not as potently as BaP. At the same DBC substrate concentration (0.17 µM), 1 µM of Supermix-10 inhibited DBC metabolism by 14% (Figure 4B). Again, BIC favored Equation (4) over Equation (5) as the best competitive inhibition model for both inhibitors of DBC metabolism (BICs: −52 vs. −45 for BaP and −67 vs. −64 for Supermix-10). The inhibition constant (*K_i_*) for BaP optimized at a ~10-fold lower value than the *K_i_* for Supermix-10, indicating that BaP is a more potent inhibitor of DBC metabolism (Table 4).

### 3.2. Interaction PBPK Modeling

**Figure 5:**
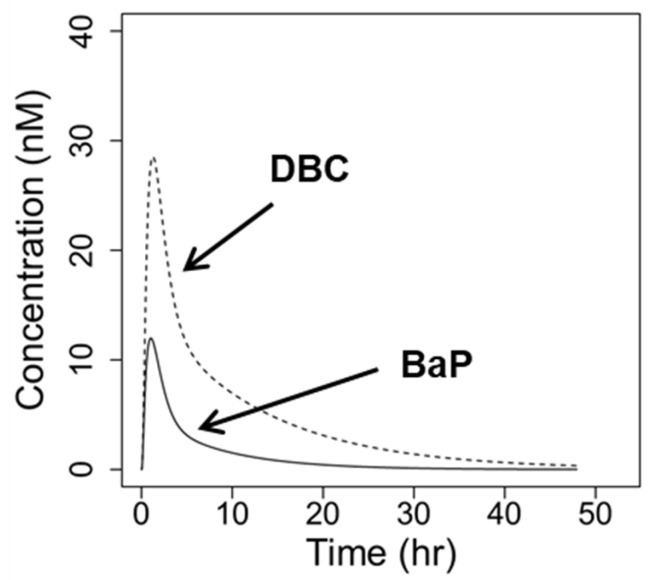
Predicted concentrations of benzo[a]pyrene (BaP, solid line) and dibenzo[def,p]chrysene (DBC, dashed line) in blood over time of a reference 73 kg human following an oral doses of 1 pmol BaP (0.25 ng) or DBC (0.30 ng), respectively.

After integrating new in vitro metabolism parameters for BaP and DBC (Table 3) in the PBPK interaction model using standard in vitro to in vivo extrapolation parameters, the model predicts higher concentrations of DBC than BaP in blood at equivalent molar doses. Assuming no competitive inhibition (i.e., *K_i_* values set to 1 × 10^99^ µM) and oral doses of 1 pmol BaP (0.25 ng) or DBC (0.3 ng), the PBPK model predicts two-fold lower peak concentrations of BaP than DBC in blood (12.0 vs. 28.6 nM, respectively), with DBC exhibiting a nearly four-fold higher AUC compared to BaP (Figure 5). Faster human metabolisms of BaP compared to DBC drive these predicted differences in parent PAH residence time, which are consistent with observed pharmacokinetic data in humans [25,26,27,28].

**Figure 6:**
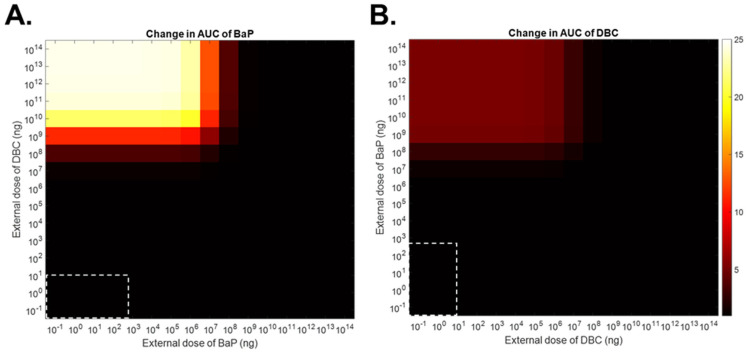
Predicted change in area under the curve (AUC) values of concentrations of benzo[a]pyrene (BaP, (**A**)) and dibenzo[def,p]chrysene (DBC, (**B**)) in blood over time following oral co-exposures to BaP and DBC at various doses. Change in AUC is calculated as a ratio of AUCs assuming competitive inhibition of metabolism (i.e., measured inhibition coefficients (*K_i_*)) to AUCs assuming no competitive inhibition of metabolism (i.e., very high *K_i_*). White dotted boxes indicate ranges of typical human exposures to BaP and DBC [28,29,32].

When assuming competitive inhibition during co-exposures of BaP and DBC, the PBPK interaction model predicts significant increases in AUCs of concentrations of BaP and DBC in blood at high PAH doses. For example, when considering DBC inhibiting BaP metabolism, the PBPK interaction model predicts that DBC oral doses ≥100 mg will increase AUCs of BaP concentration in blood at BaP doses ≤100 mg (Figure 6A). The model predicts that at higher doses of BaP (≥1 g), sufficient internal concentration of BaP will outcompete DBC as an inhibitor at concentrations simulated, as indicated by no significant change in the AUC of BaP concentration in blood (Figure 6A). Similarly, when considering BaP inhibiting DBC metabolism, BaP oral doses ≥100 mg increased AUCs of DBC concentration in blood at DBC doses ≤10 mg (Figure 6B). However, at DBC doses ≥100 mg, no significant change occurred in the AUC of DBC concentration in blood, indicating sufficiently high concentrations of DBC minimizes competitive metabolic inhibition by BaP (Figure 6B).

The PBPK interaction model predicts that DBC will have a more potent effect on internal BaP concentrations (up to a 25-fold increase in the AUC of the BaP concentration in blood) than BaP altering internal DBC concentrations (up to a 5-fold increase in the AUC of the DBC concentration in blood) (Figure 6). This observation is surprising considering that inhibition coefficients measured in vitro favor BaP as a more potent inhibitor of DBC metabolism than DBC of BaP metabolism (*K_i_* 0.061 vs. 0.44 µM, respectively). This observation suggests that rates of substrate metabolism also play a significant role in determining the magnitude of impact on internal dose metrics of competitively inhibiting metabolism. Here, the intrinsic clearance of BaP is nearly five-fold faster than that of DBC (Table 3). Thus, inhibiting BaP metabolism has greater effect on the BaP concentration in the blood AUC compared to inhibiting the slower DBC metabolism and resulting effect on the DBC concentration in the blood AUC.

## 4. Discussion

To quantitatively understand implications of competitive metabolism of PAHs, we measured competitive inhibition coefficients of BaP and DBC on each other’s metabolism in vitro using human hepatic microsomes. We developed a PBPK interaction model to translate these measurements made in vitro and predicted internal PAH dosimetry in scenarios of binary PAH exposures to humans. Finally, we measured inhibition coefficients of Supermix-10 on BaP and DBC metabolism to assess the potential of competitive metabolism by an environmental mixture.

The current study represents the first efforts to utilize this approach to assess the competitive metabolism of PAHs from environmental exposures. In the past, other researchers have employed similar approaches to evaluate the impact of completive metabolism on the internal dosimetry of chemicals and drugs. For chemical risk, toxicologists have measured in vitro competitive metabolism and integrated measured parameters into PBPK models to evaluate implications of exposures to binary and more complex mixtures of volatile organic chemicals such as dichloromethane, benzene, toluene, ethylbenzene, and xylene [33,34,35]. More recently, pharmaceutical developers have used this approach to evaluate drug–drug interactions during drug development [36,37,38]. In a recently released clinical drug interaction guidance, the US Food and Drug Administration (FDA) states that PBPK models can be used in lieu of some prospective drug–drug interaction studies and cites several successful PBPK predictions of weak to moderate CYP inhibitors [39]. This study demonstrates the utility of this approach for assessing the risk of chemicals found at Superfund Sites.

Measuring inhibition in human microsomes provides both advantages and limitations compared to other model systems such as isolated enzymes. Derived from liver tissues, hepatic microsomes offer a full complement of CYPs and other enzymes found at proportional levels to in vivo systems. As such, microsomes are commonly used for in vitro metabolism studies, and in vitro to in vivo extrapolation methods are well defined [40]. One limitation of using microsomes for metabolism measurements is that the inhibition coefficients measured here are apparent due to the presence of several enzymes, including CYPs involved with BaP and DBC metabolism, all capable of metabolizing the various PAHs. Theoretically, each enzyme will have an individual inhibition coefficient for each PAH inhibitor. As such, the composite inhibition coefficient measured here provides an apparent inhibition coefficient most applicable at substrate concentrations measured, which, here, was ~0.17 µM BaP or DBC. Measuring at lower concentrations would challenge analytical detection limits and our ability to reliably measure competitive inhibition.

The PBPK interaction model predicts significant increases in PAH concentration in blood AUCs during PAH co-exposures but only at BaP and DBC exposures much higher than those experienced by the general population. Few have reported exposure assessments for DBC; however, Madeen et al. estimates 9 ng/d oral intake of DBC for a 70 kg nonsmoking adult based on the French diet of 2013 [26]. Presumably, smokers and those consuming diets containing more smoked foods would have higher exposures to DBC. For BaP, the EPA estimates that a non-smoking adult in the U.S. would be exposed to 250–750 ng/d BaP [41]. In comparison, the PBPK interaction model predicts that much higher PAH doses (≥100 mg) are required to cause increases in PAH concentration in blood AUC values due to competitive metabolism, five orders of magnitude higher than reported human exposures. A critical assumption of the Relative Potency Factor approach assumes that exposure to mixtures will not cause interactions among mixture components at typical human exposures [9]. The PBPK interaction model predictions for BaP and DBC align well with this assumption.

To begin to answer the question of whether competitive inhibition is a concern with PAHs found at higher levels in the environment, we measured competitive inhibition by Supermix-10 on BaP and DBC metabolism. Supermix-10 contains the top 10 most abundantly measured PAHs at the Portland Harbor Superfund site measured in 2010 and 2015 [20,32]. Treating a mixture of 10 chemicals as a single inhibitor is an obvious simplification of the more complex system. In order to more fully characterize the metabolic interactions of the complete system, a sub-model for each compound would need to be developed (10 additional PBPK models), and inhibition coefficients for each potential metabolic interaction would need to be measured in a factorial design. This creates a major roadblock to investigating competitive inhibition with increasingly complicated environmental mixtures. While higher-throughput techniques may exist in the future to make this evaluation less resource-intensive, at present, we began to address this issue here by using a more pragmatic, simplified approach.

Inhibition coefficients for Supermix-10 inhibiting BaP and DBC metabolism were very similar to those of DBC and BaP, respectively. With respect to BaP metabolism, Supermix-10 inhibition coefficients were within 95% confidence intervals of DBC, and with respect to the DBC metabolism, BaP was a ~10-fold more potent inhibitor compared to Supermix-10 (Table 4). Human exposures to Supermix-10 have not been highly characterized; however, studies (including Portland Harbor Public Health Assessment) have estimated angler exposure to fish from the Portland Harbor Superfund site [32,42]. Using measured PAH concentrations in fish or passive sampling devices, as well as 90th and 99th percentile fish consumption rates, Supermix-10 exposures have been estimated to range from 1.4 to 84 µg/d [32,42]. These estimated Supermix-10 exposures for a high-exposure subpopulation are still three orders of magnitude below BaP and DBC exposures predicted to impact BaP or DBC pharmacokinetics. Of course, measuring pharmacokinetic properties (e.g., metabolism, inhibition coefficients) of individual Supermix-10 compounds (particularly PAHs with the highest environmental levels and toxicity), developing computational models, and predicting internal dosimetry of Supermix-10 constituents would offer a more accurate assessment of the effects of competitive metabolism following combined exposures to BaP, DBC, and Supermix-10.

An additional extension of this approach could evaluate the impact of metabolites (e.g., benzo[a]pyrene-7,8-dihydrodiol, dibenzo[def,p]chrysene-11,12 diol, etc.) on competitive metabolism, particularly of the ultimate toxicant. CYPs and Phase II enzymes can metabolize newly formed, first-generation PAH metabolites [29,30], creating competition with other substrates in the system (such as the parent PAH). Newly formed second-generation metabolites can also enter the increasingly complex system of competition as additional substrates compete for important enzymes. Researchers have noted direct and indirect evidence that BaP metabolites inhibit BaP metabolism, which in the case of competitive metabolism, is also true vice versa [31]. Since it is extremely difficult to remove new PAH metabolites as they are formed, metabolism measured here by the rate of disappearance of the parent PAH has these interactions somewhat factored into the measured apparent metabolism rates. Allowing metabolites to exist concurrently offers a more realistic model to the in vivo environment compared to the herculean task of attempting to purify the system of newly formed metabolite inhibitors. In this respect, microsome systems offering a full complement of relevant enzymes serves as a better model system for competitive inhibition evaluation compared to a model system of individual purified enzymes. Other situations, such as enzyme induction after repeated PAH exposures, can further complicate these evaluations [31].

## 5. Conclusions

We developed an approach to assess implications of competitive metabolism by PAHs. We quantitatively measured competitive inhibition of BaP and DBC metabolism by BaP, DBC, and Supermix-10 in human liver microsomes. Using those in vitro measurements, we developed an interaction PBPK model for BaP and DBC. The interaction PBPK model predicts that human doses of BaP and DBC required to impact internal dose metrics are much higher than typical human exposures. This prediction is consistent with assumptions in the Relative Potency Factor approach. This approach used here provides a framework that could be further extended to assess whether Relative Potency Factor approach assumptions hold true for other PAHs and PAH metabolites of concern at Superfund Sites, particularly PAHs found at higher levels in the environmental. Verifying critical assumptions is a crucial step for successfully applying the Relative Potency Factor approach for the risk assessment of complex mixtures.

## Figures and Tables

**Table 1:** Polycyclic aromatic hydrocarbons (PAHs) found in Supermix-10. Reported molar ratios are normalized to 2-methylnapthalene, the lowest abundant PAH in the mixture.

Compound	CAS	Molecular Weight	Molar Ratio	Molar Fraction	Carcinogenic ^A^
		(g/mol)			
benzo(a)anthracene	56-55-3	228.29	2.47	0.05	EPA, IARC, ACGIH
retene	483-65-8	234.34	7.43	0.15	
pyrene	129-00-0	202.25	14.50	0.28	
phenanthrene	85-01-8	178.23	1.72	0.03	
naphthalene	91-20-3	128.17	3.48	0.07	EPA, IARC, ACGIH
fluorene	86-73-7	166.22	1.84	0.04	
fluoranthene	206-44-0	202.25	14.24	0.28	
chrysene	218-01-9	228.29	2.59	0.05	EPA, ACGIH
acenaphthylene	208-96-8	154.21	2.00	0.04	
2-methylnaphthalene	91-57-6	142.20	1.00	0.02	

^A^ Classified as Suspected, Possible, Probable, or Confirmed carcinogen in animal models or humans by United States Environmental Protection Agency (EPA), International Agency for Research on Cancer (IARC), or American Conference of Governmental Industrial Hygienists (ACGIH).

**Table 2:** Bayesian information criteria (BIC) for metabolism model fits to rates of benzo[a]pyrene (BaP) and dibenzo[def,p]chrysene (DBC) metabolism in human hepatic microsomes at different substrate concentrations.

Model	BIC	
	BaP	DBC
Michaelis-Menten	–71.1	–71.6
Michaelis-Menten-clearance	–73.5	–88.3
Double Michaelis-Menten	–71.5	–86.8

**Table 3:** Metabolic parameters of benzo[a]pyrene (BaP) and dibenzo[def,p]chrysene (DBC) metabolism in human hepatic microsomes fit to the Michaelis–Menten clearance model, including 95% confidence intervals (CI).

Compound	Parameter							
	Vmax_1_		*Km*_1_		*Cl_int_*_1_		*Cl_int_*_2_	
	nmol/min/mg Micro	95% CI	µM	95% CI	mL/min	95% CI	mL/min	95% CI
BaP	0.0063	0.0044–0.0083	0.088	0.044–0.15	0.072	0.054–0.10	0.0012	1.7 × 10^−7^–0.0028
DBC	0.00090	0.00044–0.0023	0.060	0.014–0.22	0.015	0.0097–0.032	0.0017	6.5 × 10^−7^–0.0028

**Table 4:** Inhibition constants (*K_i_*) with 95% confidence intervals (CI) of competitive inhibitors to benzo[a]pyrene (BaP) and dibenzo[def,p]chrysene (DBC) metabolism in human hepatic microsomes. Inhibitors included BaP, DBC, and Supermix-10 (SM10).

Substrate	Inhibitor					
	*K_i_*_BaP_		*K_i_*_DBC_		*K_i_*_SM10_	
	µM	95% CI	µM	95% CI	µM	95% CI
BaP			0.44	0.36–0.54	0.75	0.49–1.1
DBC	0.061	0.041–0.12			0.63	0.36–1.2
